# Loss to Follow-Up in Patients With Proliferative Diabetic Retinopathy or Diabetic Macular Edema

**DOI:** 10.1001/jamanetworkopen.2024.50942

**Published:** 2024-12-13

**Authors:** Ryan S. Huang, Sumana C. Naidu, Andrew Mihalache, Marko M. Popovic, Peter J. Kertes, David Sarraf, SriniVas R. Sadda, Rajeev H. Muni, Radha P. Kohly

**Affiliations:** 1 Temerty Faculty of Medicine, University of Toronto, Toronto, Ontario, Canada; 2 Department of Ophthalmology and Vision Sciences, University of Toronto, Toronto, Ontario, Canada; 3 Stein Eye Institute, David Geffen School of Medicine, University of California, Los Angeles; 4 Doheny Eye Institute, David Geffen School of Medicine, University of California, Los Angeles; 5 John and Liz Tory Eye Centre, Sunnybrook Health Sciences Centre, Toronto, Ontario, Canada; 6 Department of Ophthalmology, St. Michael’s Hospital/Unity Health Toronto, Toronto, Ontario, Canada

## Abstract

**Importance:**

Effective management of proliferative diabetic retinopathy (PDR) and diabetic macular edema (DME) requires reliable patient follow-up to prevent disease progression.

**Objective:**

To investigate the sociodemographic and clinical factors associated with being lost to follow-up (LTFU) among individuals with PDR or DME treated with anti–vascular endothelial growth factor (VEGF) intravitreal injections (IVIs) or panretinal photocoagulation (PRP).

**Design, Setting, and Participants:**

This cohort study included a multicenter, retrospective review of patients with PDR or DME treated in Toronto, Canada, from January 1, 2012, to December 31, 2021. Data were analyzed from February 1 to May 31, 2024.

**Exposures:**

All patients received at least 1 anti-VEGF IVI or PRP session.

**Main Outcomes and Measures:**

The primary outcome was the LTFU rate, defined as the absence of an ophthalmic visit or intervention in the 1-year period following an individual’s last visit with the treating retinal specialist. Univariable and multivariable logistic regression models were conducted to evaluate associations between sociodemographic and clinical factors with the LTFU rate.

**Results:**

Overall, 2961 patients with PDR or DME (mean [SD] age, 71 [13] years; 1640 [55.4%] male) were included, of whom 507 (17.1%) were LTFU over a mean (SD) follow-up period of 61 (22) months. In the multivariable analysis, older patients (age ≥85 years vs age <65 years: odds ratio [OR], 0.58; 95% CI, 0.40-0.81; *P* = .002), those with worse baseline visual acuity (>20/200 Snellen vs 20/40 Snellen or better: OR, 0.68; 95% CI, 0.48-0.97; *P* = .04), those with DME (OR vs no DME, 0.60; 95% CI, 0.43-0.83; *P* = .003), those with frequent clinic visits (≥6 visits vs <6 visits: OR, 0.78; 95% CI, 0.62-0.98; *P* = .04), and those with a high anti-VEGF IVI burden in the first year (OR vs low anti-VEGF burden, 0.40; 95% CI, 0.21-0.76; *P* = .006) were less likely to be LTFU. In contrast, males (OR vs females, 1.23; 95% CI, 1.04-1.52; *P* = .04), patients living further from the point of care (>200 vs ≤20 km OR, 2.65; 95% CI, 1.85-3.76; *P* < .001), and those treated with PRP (OR vs anti-VEGF IVIs, 2.10; 95% CI, 1.24-3.55; *P* < .001) were more likely to be LTFU. Compared with White patients, Black patients (OR, 2.10; 95% CI, 1.50-2.95; *P* < .001) and Hispanic patients (OR, 1.54; 95% CI, 1.05-2.21; *P* = .03) were more likely to be LTFU.

**Conclusions and Relevance:**

This cohort study found multiple factors associated with LTFU rates. Identifying individuals at higher risk of LTFU and developing targeted strategies may reduce disease progression and vision loss in individuals with PDR.

## Key Points

**Question:**

What is the lost to follow-up (LTFU) rate of patients receiving treatment for proliferative diabetic retinopathy or diabetic macular edema and what factors are associated with LTFU rates?

**Findings:**

In this cohort study of 2961 patients with proliferative diabetic retinopathy or diabetic macular edema, 17% were LTFU. Higher odds of LTFU were found among males, patients living further from the point of care, patients who were Black or Hispanic, patients receiving panretinal photocoagulation (vs anti–vascular endothelial growth factor intravitreal injections), and patients with lower intravitreal injection burden.

**Meaning:**

The findings of this study identified risk factors associated with LTFU that could help to inform targeted strategies to reduce these rates.

## Teaser

This cohort study investigates the sociodemographic and clinical factors associated with being lost to follow-up among individuals with proliferative diabetic retinopathy and diabetic macular edema treated at 2 clinics in Toronto, Ontario, Canada.

## Introduction

Diabetic retinopathy (DR) is the most prevalent microvascular complication of diabetes and a leading cause of vision loss globally.^1^ The most common cause of vision loss is diabetic macular edema (DME), which can occur in the nonproliferative form of DR and the proliferative form of DR (PDR). Intravitreal injections (IVIs) of anti–vascular endothelial growth factor (VEGF) agents are widely administered as a primary treatment for patients with DME or as a primary or adjunctive therapy to panretinal photocoagulation (PRP) in patients with PDR.^2,3^ In the setting of DME, anti-VEGF IVIs may substantially reduce the risk of vision loss and may improve visual acuity.^4,5^

The management of DR necessitates frequent clinic visits, hence reliable patient follow-up is paramount to improving functional and anatomical outcomes.^6^ Interruptions in treatment regimens may exacerbate DR progression and contribute to irreversible vision loss. It is important for clinicians to understand the factors associated with patients being lost to follow-up (LTFU).^6,7,8^ It is estimated that 12% to 61% of patients with PDR may become LTFU, which may be attributed to a complex interplay among social determinants of health.^7,8,9,10,11,12^ Further understanding of the risk factors associated with higher LTFU rates is critical to develop corresponding measures that can be integrated into the care of patients with DR. Our investigation aims to determine associations of sociodemographic and clinical factors with LTFU rates in patients with PDR or DME.

## Methods

This cohort study was approved by the Sunnybrook Research Ethics Board and the Unity Health Toronto Research Ethics Board and adhered to the tenets of the Declaration of Helsinki. Informed consent was not required for this study, as it involved secondary use of deidentified data in adherence with the Tri-Council Policy Statement: Ethical Conduct for Research Involving Humans. This study is reported following the Strengthening the Reporting of Observational Studies in Epidemiology (STROBE) reporting guideline.

### Study Design and Eligibility

We conducted a multicenter, retrospective review using electronic medical record (EMR) data for all patients with PDR or DME requiring treatment from 2 retina specialists (R.H.M and R.P.K.) at St. Michael’s Hospital (which had 7 full-time retina specialists at the time this study was conducted) and Sunnybrook Health Sciences Centre (which had 3 full-time retina specialists at the time this study was conducted), in Toronto, Canada, between January 1, 2012, and December 31, 2021. Consecutive patients from both specialists were included, ensuring that all patients who met the selection criteria were recruited from both specialists and that the patient sample was representative of the broader patient population at each clinical site. Our inclusion criteria were patients aged at least 18 years, patients receiving at least 1 PRP treatment or anti-VEGF IVI for PDR or DME over the duration of follow-up, and patients with at least 1 year of follow-up with their retina specialist after their initial consultation, which represented the exposure assessment period beginning at the initial consultation visit and lasting 1 year. Patients with initial nonproliferative DR were included if they subsequently developed PDR or DME and required treatment. We excluded patients who did not require treatment for the entire duration of follow-up, as well as those who were referred to another retina specialist for follow-up and treatment.

### Data Collection

Patients with an initial consultation for retinal vascular disease were screened for inclusion using Ontario Health Insurance Plan (OHIP) billing codes based on the treatment received: PRP (E154) or anti-VEGF IVI (E149). The following variables were extracted from eligible patients at every patient visit during the study period: age, sex, study eye, distance from primary residence to ophthalmology point of care in kilometers based on postal code or home address, lens status, ocular history, best corrected visual acuity (BCVA), presence of PDR, DME, neovascularization of the iris (NVI), and tractional retinal detachment, as well as the type of treatment administered (ie, PRP or anti-VEGF IVI). Presumed race and ethnicity were determined using bayesian improved surname geocoding via the R package wru, which leverages the 2010 US Census surname dataset containing more than 160 000 names.^13^ Race and ethnicity were categorized as Asian, Black, Hispanic, White, and other (eg, mixed race or those not fitting into predefined categories). Race and ethnicity were assessed to examine potential disparities in LTFU rates. With respect to treatment modalities, patients receiving fewer than 6 anti-VEGF IVIs over the 1-year exposure period were categorized as having a low injection burden, and those receiving 6 or more anti-VEGF IVIs were categorized as having a high injection burden. Moreover, patients receiving fewer than 4 PRP sessions within the 1-year exposure period were categorized as having a low PRP treatment burden, and those receiving 4 or more PRP sessions were categorized as having a high PRP treatment burden.

### Outcomes

Our primary outcome was the LTFU rate, defined as the absence of an ophthalmic visit or intervention in the 1-year period following a patient’s last visit to their treating retina specialist. Patients who were LTFU were further categorized as being temporarily LTFU or permanently LTFU, depending on whether a subsequent visit was recorded following the minimum 1-year period with no visits or interventions. Our secondary outcomes pertained to associations between LTFU rates and patient characteristics, including baseline age, sex, distance from primary residence to the point of care in kilometers, lens status, BCVA, presence of PDR or DME, and the type of treatment.

### Statistical Analysis

We conducted statistical analyses using Stata version 17.0 (StataCorp) and reported descriptive statistics for all variables. Normally distributed data were reported as means and SDs. Univariable and multivariable logistic regressions were performed to elicit associations of LTFU rates with sociodemographic and clinical characteristics, accompanied by odds ratios (ORs). Variables with either global or individual category significance (*P* < .20) were included in the multivariable model. These variables were age, sex, race and ethnicity, distance, baseline BCVA, baseline DME, treatment at the first visit, treatment type at the first visit, number of visits in the first year, number of IVIs in the first year, and number of PRP sessions in the first year for our primary analysis. Patients with missing, unknown, or indeterminate values for any covariate were excluded from the multivariable regression model. Additionally, we performed exploratory multivariable analyses incorporating interaction terms based on clinically relevant hypotheses. Secondary analyses were performed to examine associations in patients who became either temporarily or permanently LTFU. Subgroup analyses were conducted using lower thresholds for IVI and PRP burden (ie, ≥3 IVIs or ≥2 PRP sessions over the 1-year exposure period). A sensitivity analysis of treatment-related outcomes was conducted using a 1-year exposure assessment period beginning at the initial treatment instead of the initial visit. We considered 2-sided *P* < .05 statistically significant and reported 95% CIs for all analyses. Data were analyzed from February 1 to May 31, 2024.

## Results

### Demographic Characteristics

The study cohort consisted of 2961 patients with DR receiving anti-VEGF IVIs or PRP (mean [SD] age, 71 [13] years; 1640 [55.4%] male) who were followed for a mean (SD) of 61 (22) months (Figure and Table 1). Most patients were phakic (2298 patients [77.6%]). There were 665 Asian patients (22.5%), 203 Black patients (6.9%), 180 Hispanic patients (6.1%), 1848 White patients (62.4%), and 65 patients (2.2%) of another race or ethnicity. Of 2961 patients with DR, 507 (17.1%) became LTFU following the 1-year exposure period. Among them, 276 patients (54.4%) were temporarily LTFU and 231 patients (45.6%) were permanently LTFU (Table 2).

**Figure.**
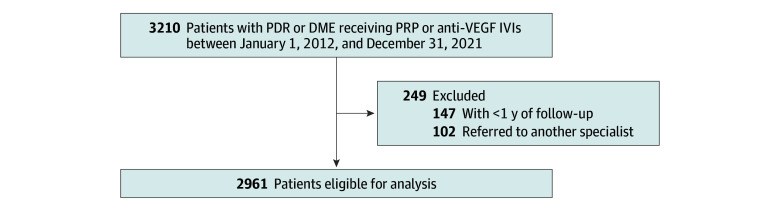
Participant Selection Flowchart DME indicates diabetic macular edema; IVI, intravitreal injection; PDR, proliferative diabetic retinopathy; PRP, panretinal photocoagulation; VEGF, vascular endothelial growth factor.

**Table 1.** Demographic and Clinical Characteristics of Study Patients

Characteristic	Patients, No. (%)^a^		
	Overall (N = 2961)	Not LTFU (n = 2454)	LTFU (n = 507)
Age, y			
Mean (SD)	71.0 (13.0)	72.2 (13.2)	68.6 (13.6)
<65	845 (28.5)	672 (79.5)	173 (20.5)
65-74	899 (30.4)	745 (82.9)	154 (17.1)
75-84	805 (27.2)	681 (84.6)	124 (15.4)
≥85	412 (13.9)	356 (86.4)	56 (13.6)
Sex			
Female	1321 (44.6)	1115 (84.4)	206 (15.6)
Male	1640 (55.4)	1339 (81.6)	301 (18.4)
Race			
Asian	665 (22.5)	552 (83.0)	113 (17.0)
Black	203 (6.9)	146 (71.9)	57 (28.1)
Hispanic	180 (6.1)	140 (77.8)	40 (22.2)
White	1848 (62.4)	1563 (84.6)	285 (15.4)
Other	65 (2.2)	53 (81.5)	12 (18.5)
Distance to place of care, km^b^			
<20	1215 (43.7)	1079 (88.8)	136 (11.2)
20-34	685 (24.6)	545 (79.6)	140 (20.4)
35-49	538 (19.4)	432 (80.3)	106 (19.7)
50-200	238 (8.6)	187 (78.6)	51 (21.4)
>200	103 (3.7)	73 (70.9)	30 (29.1)
Lens status			
Phakic	2298 (77.6)	1900 (82.7)	398 (17.3)
Pseudophakic	663 (22.4)	554 (83.6)	109 (16.4)
Baseline visual acuity			
≤20/40	801 (27.1)	632 (78.9)	169 (21.1)
20/50-20/200	1822 (61.5)	1500 (82.3)	322 (17.7)
>20/200	338 (11.4)	289 (85.5)	49 (14.5)
Baseline characteristics			
PDR	223 (7.5)	181 (81.2)	42 (18.8)
DME	331 (11.2)	292 (88.2)	39 (11.8)
NVI	56 (1.9)	48 (85.7)	8 (14.2)
Tractional RD	50 (1.7)	41 (82.0)	9 (18.0)
Treatment at first visit	520 (17.6)	442 (85.0)	78 (15.0)
Treatment type at first visit^c^			
Anti-VEGF	295 (56.7)	266 (90.2)	29 (9.8)
PRP	215 (41.3)	171 (79.5)	44 (20.5)
Both	10 (1.9)	5 (50.0)	5 (50.0)
Visits in first year, No.			
<6	2191 (74.0)	1798 (82.1)	393 (17.9)
≥6	770 (26.0)	656 (85.2)	114 (14.8)
IVI in first year, No.			
<6	2840 (95.9)	2342 (82.5)	498 (17.5)
≥6	121 (4.1)	112 (92.6)	9 (7.4)
PRP in first year, No.			
<4	2693 (90.9)	2240 (83.2)	453 (16.8)
≥4	268 (9.1)	214 (79.9)	54 (20.1)

Abbreviations: DME, diabetic macular edema; LTFU, lost to follow up; IVI, intravitreal injection; NVI, neovascularization of the iris; PDR, proliferative diabetic retinopathy; PRP, panretinal photocoagulation; RD, retinal detachment; VEGF, vascular endothelial growth factor.

^a^ Overall percentages are given for the column. Percentages for LTFU and not LTFU are given for the row.

^b^ Address data were available for 2779 patients.

^c^ Does not sum to total since only considering patients receiving treatment at first visit.

**Table 2.** Demographic and Clinical Characteristics of Patients Permanently vs Temporarily LTFU

Characteristic	Patients, No. (%)	
	Permanent LTFU (n = 231)	Temporary LTFU (n = 276)
Age, y		
<65	68 (39.3)	105 (60.7)
65-74	59 (38.3)	95 (61.7)
75-84	69 (55.6)	55 (44.4)
≥85	35 (62.5)	21 (37.5)
Sex		
Female	92 (44.7)	114 (55.3)
Male	139 (46.2)	162 (53.8)
Race		
Asian	44 (38.9)	69 (61.1)
Black	43 (75.4)	14 (24.6)
Hispanic	28 (70.0)	12 (30.0)
White	112 (39.3)	173 (60.7)
Other	4 (33.3)	8 (66.7)
Distance to place of care, km		
<20	52 (38.2)	84 (61.8)
20-34	63 (45.0)	77 (55.0)
35-49	48 (45.3)	58 (54.7)
50-200	28 (54.9)	23 (45.1)
>200	13 (43.3)	17 (56.7)
Lens status		
Phakic	168 (42.2)	230 (57.8)
Pseudophakic	63 (57.8)	46 (42.2)
Baseline visual acuity		
≤20/40	74 (43.8)	95 (56.2)
20/50-20/200	153 (47.5)	169 (52.5)
>20/200	26 (53.1)	23 (46.9)
Baseline characteristics		
PDR	14 (33.3)	28 (66.7)
DME	16 (41.0)	23 (59.0)
NVI	2 (25.0)	6 (75.0)
Tractional RD	3 (33.3)	6 (66.7)
Treatment at first visit	30 (38.5)	48 (61.5)
Treatment type at first visit		
Anti-VEGF	12 (41.4)	17 (58.6)
PRP	16 (36.4)	28 (63.6)
Both	2 (40.0)	3 (60.0)
Visits in first year, No.		
<6	171 (43.5)	222 (56.5)
≥6	60 (52.6)	54 (47.4)
IVI in first year, No.		
<6	229 (46.0)	269 (54.0)
≥6	2 (22.2)	7 (77.8)
PRP in first year, No.		
<4	215 (47.5)	238 (52.5)
≥4	16 (29.6)	38 (70.4)

Abbreviations: DME, diabetic macular edema; IVI, intravitreal injection; LTFU, lost to follow-up; NVI, neovascularization of the iris; PDR, proliferative diabetic retinopathy; PRP, panretinal photocoagulation; RD, retinal detachment; VEGF, vascular endothelial growth factor.

### Multivariable Regression

In multivariable analysis, compared with patients who were younger than 65 years, those aged 75 to 84 years (OR, 0.72; 95% CI, 0.55-0.95; *P* = .02) or 85 years or older (OR, 0.58; 95% CI, 0.40-0.81; *P* = .002) had lower odds of being LTFU (Table 3). Patients with a baseline BCVA worse than 20/200 Snellen had lower odds of being LTFU compared with patients presenting with a BCVA of 20/40 Snellen or better (OR, 0.68; 95% CI, 0.48-0.97; *P* = .04). Patients with DME at baseline had lower odds of being LTFU (OR, 0.60; 95% CI, 0.43-0.83; *P* = .003), while no significant association was observed in patients with PDR, NVI, or tractional retinal detachment at baseline (Table 3). Compared with patients who visited the clinic fewer than 6 times during their first year, patients with 6 or more clinic visits had a decreased odds of being LTFU (OR, 0.78; 95% CI, 0.62-0.98; *P* = .04). Similarly, patients with a high anti-VEGF IVI burden during their initial year of follow-up had lower odds of being LTFU compared with those with a low anti-VEGF IVI burden during their initial year (OR, 0.40; 95% CI, 0.21-0.76; *P* = .006). These findings were consistent in a subgroup analysis with a threshold of at least 3 IVIs in the first year (OR, 0.53; 95% CI, 0.35-0.81; *P* = .01).

**Table 3.** Univariable and Multivariable Regression Results for Odds of Loss to Follow-Up

Characteristic	Unadjusted OR (95% CI) (N =2961)	*P* value	Adjusted OR (95% CI) (n = 2779)^a^	*P* value
Age, y				
<65	1 [Reference]	NA	1 [Reference]	NA
65-74	0.80 (0.63-1.02)	.07	0.85 (0.65-1.10)	.11
75-84	0.71 (0.55-0.91)	.008	0.72 (0.55-0.95)	.02
≥85	0.61 (0.44-0.85)	.003	0.58 (0.40-0.81)	.002
Sex				
Female	1 [Reference]	NA	1 [Reference]	NA
Male	1.22 (1.01-1.48)	.049	1.23 (1.04-1.52)	.04
Race				
Asian	1.12 (0.88-1.43)	.34	1.08 (0.84-1.40)	.50
Black	2.14 (1.54-2.98)	<.001	2.10 (1.50-2.95)	<.001
Hispanic	1.57 (1.08-2.28)	.02	1.54 (1.05-2.21)	.03
White	1 [Reference]	NA	1 [Reference]	NA
Other	1.27 (0.67-2.40)	.47	1.18 (0.65-2.12)	.47
Distance to place of care, km				
<20	1 [Reference]	NA	1 [Reference]	NA
20-34	1.99 (1.53-2.86)	<.001	1.91 (1.41-2.58)	<.001
35-49	1.94 (1.47-2.56)	<.001	1.85 (1.35-2.51)	<.001
50-200	2.16 (1.51-3.09)	<.001	2.05 (1.42-2.96)	<.001
>200	2.78 (2.02-3.83)	<.001	2.65 (1.85-3.76)	<.001
Lens status				
Phakic	1 [Reference]	NA	NA	NA
Pseudophakic	0.94 (0.74-1.18)	.60	NA	NA
Baseline visual acuity				
≤20/40	1 [Reference]	NA	1 [Reference]	NA
20/50-20/200	0.78 (0.57-1.08)	.13	0.72 (0.50-1.03)	.08
>20/200	0.75 (0.56-0.99)	.047	0.68 (0.48-0.97)	.04
Baseline PDR				
No	1 [Reference]	NA	NA	NA
Yes	1.13 (0.80-1.61)	.481	NA	NA
Baseline DME				
No	1 [Reference]	NA	1 [Reference]	NA
Yes	0.62 (0.44-0.87)	.007	0.60 (0.43-0.83)	.003
Baseline NVI				
No	1 [Reference]	NA	NA	NA
Yes	0.80 (0.38-1.71)	.57	NA	NA
Baseline tractional RD				
No	1 [Reference]	NA	NA	NA
Yes	1.06 (0.51-2.20)	.87	NA	NA
Treatment at first visit				
No	1 [Reference]	NA	1 [Reference]	NA
Yes	0.83 (0.64-1.08)	.16	0.87 (0.61-1.18)	.34
Treatment type at first visit				
IVI	1 [Reference]	NA	1 [Reference]	NA
PRP	2.36 (1.42-3.92)	<.001	2.10 (1.24-3.55)	<.001
Visits in first year, No.				
<6	1 [Reference]	NA	1 [Reference]	NA
≥6	0.80 (0.63-1.00)	.048	0.78 (0.62-0.98)	.04
IVI in first year, No.				
<6	1 [Reference]	NA	1 [Reference]	NA
≥6	0.38 (0.19-0.75)	.005	0.40 (0.21-0.76)	.006
PRP in first year, No.				
<4	1 [Reference]	NA	1 [Reference]	NA
≥4	1.25 (0.91-1.71)	.17	1.20 (0.86-1.67)	.27

Abbreviations: DME, diabetic macular edema; IVI, intravitreal injection; NA, not applicable; NVI, neovascularization of the iris; PDR, proliferative diabetic retinopathy; PRP, panretinal photocoagulation; OR, odds ratio; RD, retinal detachment.

^a^ Multivariable regression model was adjusted for all factors with *P* < .20 on univariable analysis, including age, sex, race and ethnicity, distance, baseline BCVA, baseline DME, treatment at the first visit, treatment type at the first visit, number of visits in the first year, number of IVIs in the first year, and number of PRP sessions in the first year. Patients with missing, unknown, or indeterminate values for any covariate were excluded from the multivariable regression model.

Male patients had higher odds of being LTFU compared with female patients (OR, 1.23; 95% CI, 1.04-1.52; *P* = .04). Patients who were Black (OR, 2.10; 95% CI, 1.50-2.95; *P* < .001) or Hispanic (OR, 1.54; 95% CI, 1.05-2.21; *P* = .03) had higher odds of being LTFU compared with patients who were White. Compared with patients living within 20 km of their point of care, those living farther away were more likely to become LTFU (20-34 km: OR, 1.91; 95% CI, 1.41-2.58; *P* < .001; 35-49 km: OR, 1.85; 95% CI, 1.35, 2.51; *P* < .001; 50-200 km: OR, 2.05; 95% CI, 1.42-2.96; *P* < .001; >200 km: OR, 2.65; 95% CI, 1.85-3.76; *P* < .001). Moreover, compared with patients initially treated with an anti-VEGF IVI, those receiving PRP were at increased odds of being LTFU (OR, 2.10; 95% CI, 1.24-3.55; *P* < .001). None of the interaction terms explored in the multivariable analysis were found to be statistically significant, indicating that no effect modification was found (eTable 1 in Supplement 1).

### Temporary and Permanent LTFU

Compared with patients aged 65 years or younger, older patients (age 75-84 years: OR, 0.58; 95% CI, 0.39-0.85; *P* < .001; age ≥85 years: OR, 0.45; 95% CI, 0.29-0.69; *P* < .001) had lower odds of being temporarily LTFU. Compared with patients with fewer than 6 clinic visits, patients with 6 or more clinic visits had lower odds of being temporarily LTFU (OR, 0.60; 95% CI, 0.39-0.95; *P* = .02). Patients receiving PRP, compared with those receiving anti-VEGF IVIs at their first visit, had higher odds of being temporarily LTFU (OR, 2.45; 95% CI, 1.65-3.90; *P* < .001) (Table 4). Patients with a high burden of PRP sessions during their initial year also had higher odds of being temporarily LTFU compared with those with a low burden (OR, 1.85; 95% CI, 1.12-2.75; *P* = .02).

**Table 4.** Univariable and Multivariable Regression Results for Patients Permanently and Temporarily LTFU

Characteristic	Permanent LTFU				Temporary LTFU			
	Unadjusted OR (95% CI) (n = 507)	*P* value	Adjusted OR (95% CI) (n = 204)^a^	*P* value	Unadjusted OR (95% CI) (n = 507)	*P* value	Adjusted OR (95% CI) (n = 259)^a^	*P* value
Age, y								
<65	1 [Reference]	NA	NA	NA	1 [Reference]	NA	1 [Reference]	NA
65-74	0.80 (0.56-1.15)	.23	NA	NA	0.83 (0.62-1.12)	.22	0.88 (0.60-1.26)	.42
75-84	1.07 (0.76-1.52)	.70	NA	NA	0.52 (0.37-0.73)	<.001	0.58 (0.39-0.85)	<.001
≥85	1.06 (0.69-1.62)	.79	NA	NA	0.38 (0.23-0.61)	<.001	0.45 (0.29-0.69)	<.001
Sex								
Female	1 [Reference]	NA	1 [Reference]	NA	1 [Reference]	NA	NA	NA
Male	1.24 (0.94-1.63)	.13	1.16 (0.88-1.72)	.28	1.16 (0.9-1.49)	.25	NA	NA
Race								
Asian	1.10 (0.77-1.58)	.61	1.10 (0.75-1.60)	.59	1.12 (0.84-1.50)	.45	NA	NA
Black	4.17 (2.83-6.13)	<.001	4.10 (2.68-6.25)	<.001	0.72 (0.41-1.26)	.25	NA	NA
Hispanic	2.86 (1.83-4.46)	<.001	2.65 (1.60-4.50)	<.001	0.69 (0.38-1.27)	.23	NA	NA
White	1 [Reference]	NA	1 [Reference]	NA	1 [Reference]	NA	NA	NA
Other	1.03 (0.37-2.89)	.95	1.08 (0.42-2.79)	.88	1.38 (0.65-2.95)	.40	NA	NA
Distance to place of care, km								
<20	1 [Reference]	NA	1 [Reference]	NA	1 [Reference]	NA	1 [Reference]	NA
20-34	2.17 (1.47-3.20)	<.001	2.34 (1.55-3.68)	<.001	1.70 (1.22-2.36)	.002	1.78 (1.18-2.71)	.007
35-49	2.23 (1.49-3.35)	<.001	2.75 (1.62-4.65)	<.001	1.59 (1.12-2.27)	.01	1.65 (1.07-2.52)	.03
50-200	2.98 (1.84-4.83)	<.001	3.45 (2.05-5.81)	<.001	1.44 (0.89-2.33)	.14	1.38 (0.87-2.23)	.19
>200	3.65 (2.36-5.63)	<.001	3.94 (2.65-6.55)	<.001	1.82 (1.20-2.78)	.005	2.05 (1.32-3.19)	.003
Lens status								
Phakic	1 [Reference]	NA	1 [Reference]	NA	1 [Reference]	NA	1 [Reference]	NA
Pseudophakic	1.33 (0.98-1.80)	.07	1.48 (0.98-2.20)	.07	0.67 (0.48-0.93)	.02	0.85 (0.59-1.25)	.42
Baseline visual acuity								
≤20/40	1 [Reference]	NA	NA	NA	1 [Reference]	NA	1 [Reference]	NA
20/50-20/200	0.86 (0.55-1.34)	.50	NA	NA	0.76 (0.51-1.14)	.18	0.95 (0.65-1.40)	.80
>20/200	0.81 (0.54-1.21)	.29	NA	NA	0.74 (0.52-1.07)	.11	0.72 (0.48-1.08)	.11
Baseline PDR								
No	1 [Reference]	NA	NA	NA	1 [Reference]	NA	1 [Reference]	NA
Yes	0.78 (0.45-1.36)	.38	NA	NA	1.44 (0.95-2.19)	.09	1.38 (0.90-2.12)	.15
Baseline DME								
No	1 [Reference]	NA	1 [Reference]	NA	1 [Reference]	NA	1 [Reference]	NA
Yes	0.55 (0.33-0.93)	.03	0.50 (0.35-0.85)	.02	0.67 (0.43-1.05)	.08	0.85 (0.53-1.37)	.51
Baseline NVI								
No	1 [Reference]	NA	NA	NA	1 [Reference]	NA	NA	NA
Yes	0.44 (0.11-1.81)	.26	NA	NA	1.11 (0.47-2.63)	.81	NA	NA
Baseline tractional RD								
No	1 [Reference]	NA	NA	NA	1 [Reference]	NA	NA	NA
Yes	0.77 (0.24-2.52)	.67	NA	NA	1.30 (0.55-3.11)	.54	NA	NA
Treatment at first visit								
No	1 [Reference]	NA	1 [Reference]	NA	1 [Reference]	NA	NA	NA
Yes	0.68 (0.46-1.01)	.06	0.90 (0.60-1.35)	.51	0.95 (0.69-1.33)	.80	NA	NA
Treatment type at first visit								
IVI	1 [Reference]	NA	1 [Reference]	NA	1 [Reference]	NA	1 [Reference]	NA
PRP	2.07 (0.96-4.49)	.06	1.98 (0.88-4.14)	.35	2.56 (1.36-4.82)	.003	2.45 (1.65-3.90)	<.001
Visits in first year								
<6	1 [Reference]	NA	NA	NA	1 [Reference]	NA	1 [Reference]	NA
≥6	1.00 (0.73-1.36)	.99	NA	NA	0.67 (0.49-0.91)	.01	0.60 (0.39-0.95)	.02
IVI in first year, No.								
<6	1 [Reference]	NA	1 [Reference]	NA	1 [Reference]	NA	1 [Reference]	NA
≥6	0.19 (0.05-0.78)	.02	0.22 (0.12-0.65)	.01	0.59 (0.27-1.27)	.18	0.68 (0.30-1.45)	.28
PRP in first year, No.								
<4	1 [Reference]	NA	NA	NA	1 [Reference]	NA	1 [Reference]	NA
≥4	0.73 (0.43-1.24)	.24	NA	NA	1.70 (1.18-2.46)	.005	1.85 (1.12-2.75)	.02

Abbreviations: DME, diabetic macular edema; IVI, intravitreal injection; LTFU, lost to follow-up; NA, not applicable; NVI, neovascularization of the iris; OR, odds ratio; PDR, proliferative diabetic retinopathy; PRP, panretinal photocoagulation; RD, retinal detachment.

^a^ Multivariable regression model was adjusted for all factors with *P* < .20 on univariable analysis. The permanent LTFU model was adjusted for sex, race and ethnicity, distance to point of care, lens status, baseline DME, treatment at first visit, treatment type at first visit, and number of IVIs in first year. The temporary LTFU model was adjusted for age, distance to point of care, lens status, baseline visual acuity, baseline PDR, baseline DME, treatment type at first visit, number of visits in the first year, number of IVIs in first year, and number of PRP sessions in the first year. Patients with missing, unknown, or indeterminate values for any covariate were excluded from the multivariable regression model.

Patients with DME (OR vs no DME, 0.50; 95% CI, 0.35-0.85; *P* = .02) at baseline and patients with a high anti-VEGF IVI burden (OR vs low anti-VEGF burden, 0.22; 95% CI, 0.12-0.65; *P* = .01) had lower odds of being permanently LTFU, while Black patients (OR, 4.10; 95% CI, 2.68-6.25; *P* < .001) and Hispanic patients (OR, 2.65; 95% CI, 1.60-4.50; *P* < .001) had higher odds of being permanently LTFU than White patients. A comprehensive summary of our secondary analysis findings can be found in Table 4.

### Exposure Period Starting at Initial Treatment

The findings of our sensitivity analysis of treatment-related outcomes considering a 1-year exposure period starting at the initial treatment rather than the initial visit are summarized in eTable 2 in Supplement 1. Almost all outcomes were consistent with the primary analysis (6 of 8 outcomes [75.0%]), except for the number of PRP sessions during the exposure period, wherein patients with a high PRP burden now had higher odds of being LTFU (OR, 1.50; 95% CI, 1.08-1.97; *P* = .02), whereas this finding was not significant in the primary analysis.

## Discussion

Our analysis of patients with PDR or DME managed at 2 independent, high-volume centers in Toronto, Canada, found that 17% of patients became LTFU over the 10-year study period. Factors significantly associated with a higher LTFU rate included male sex, residing more than 20 km away from the point of care, baseline BCVA of 20/40 Snellen or better, fewer than 6 appointments per year, low injection burden (ie, <6 anti-VEGF IVIs in the first year), and receiving initial PRP (as opposed to an anti-VEGF IVI). In contrast, worse baseline BCVA and baseline DME were associated with a lower LTFU rate.

The phenomenon of patients with DR becoming LTFU has previously been studied, with observed rates varying between 12% and 61% in patients with PDR.^9,10,11^ Our analysis expands on this prior work by providing a prolonged study period of 10 years and novel associations with LTFU rates that have not been previously described in the literature, to our knowledge. Additionally, unlike studies conducted in the US in which patients were recruited based on insurance claims, our analysis examined patients in a universal, publicly funded health care system.^9,10,11^ We found that the overall LTFU rate was 17%, which is consistent with the findings of Obeid et al,^14^ in which one-quarter of patients were LTFU from a cohort of 2302 patients with PDR.^14^ Similar to previous analyses, we also found that the administration of PRP during a patient’s first visit was associated with a higher LTFU rate.^8,9,11,14^ Compared with anti-VEGF IVIs, which must be sequentially repeated, the administration of PRP may offer a more durable treatment once completed.^2^ Thus, it is possible that the administration of PRP during a patient’s first few visits may increase the likelihood of them being LTFU, particularly if follow-up visits after initial treatment are not considered necessary by the patient or if they do not perceive any deteriorations in vision post-PRP that would warrant a return to the clinic. In contrast, few patients cancel clinic appointments for anti-VEGF IVIs.^15^ PRP is also associated with a higher likelihood of vision loss than anti-VEGF IVIs, which may further contribute to increased LTFU rates in patients receiving PRP.^16^ Nonetheless, while anti-VEGF IVIs may offer an efficacy profile comparable to that of PRP in patients with PDR, their beneficial effects on preserving and improving visual acuity are strongly dependent on a patient’s adherence to follow-up appointments, as outlined by their treatment regimen.^2,3,17^ In our investigation, we found that patients with a low IVI burden and fewer than 6 clinic visits during their first year were more likely to become LTFU. As such, less frequent clinical engagement and minimal intervention may inadvertently decrease a patient’s perceived necessity for ongoing care.

Patients with a baseline BCVA of worse than 20/200 Snellen, those presenting with DME, and those with a high injection burden or 6 or more clinic visits during their first year were significantly less likely to become LTFU in our analysis. Patients with a greater severity of DR may require a more intensive treatment regimen, such as more frequent anti-VEGF IVIs, and such intensive therapy may incentivize patients to pursue regular follow-up. Less severe disease may be associated with patients becoming LTFU, as asymptomatic patients may feel a reduced urgency to attend follow-up appointments.^9^ Complementing our findings, previous studies have found that patients with DR who have longer intervals between follow-up appointments,^18^ and those with a good baseline visual acuity had greater odds of being LTFU.^11,14^

Our study also found considerably higher LTFU rates among patients living more than 20 km away from the practice of their treating retinal specialist. Some of the patients included in our analysis lived very far from their point of care, with 3.7% living more than 200 km away; longer traveled distances represent an important barrier for patients returning to their scheduled clinic appointments. Interestingly, Obeid et al^14^ found that distances to the clinic were not associated with whether patients with PDR were LTFU; however, in their retrospective study, 90% of the cohort resided within 24 miles or approximately 39 km of their ophthalmologist.^14^ In contrast, a 2020 retrospective analysis by Green et al^9^ found that patients with PDR living within 20 miles from their place of care were significantly more likely to be LTFU. However, Green et al^9^ note that their inner-city institution represents the largest safety net hospital in the New England region of the US, and patients residing in close proximity may comprise a population with more pronounced barriers to care.^9^

In our study cohort, patients younger than 65 years had notably higher LTFU rates compared with patients aged 85 years or older. Older patients may have fewer employment obligations and childcare responsibilities compared with younger patients, thus increasing their ability to attend appointments scheduled during working hours. This extends on the findings of Obeid et al,^14^ who postulated that younger patients with PDR were more likely to become LTFU because they may lack insurance coverage or have higher deductible plans.^14^ In the US, approximately 44 million people younger than 65 years old lack health insurance.^19^ In contrast, in the province of Ontario, Canada, all eligible citizens receive health insurance coverage through the universal, publicly funded OHIP.^20^ Nevertheless, anti-VEGF IVIs are only covered by OHIP for patients aged at least 65 years and other special groups.^21^ Therefore, patients younger than 65 years receiving care at our centers may need to cover some portion of the cost of their anti-VEGF IVIs, most commonly via private insurance. They also have access to off-label bevacizumab, which is considerably cheaper for younger patients who do not have insurance. Hence, a lack of drug coverage in younger patients may not necessarily serve as a crucial barrier to attending necessary follow-up appointments.

Using bayesian improved surname geocoding, we found that Black and Hispanic patients were more likely to be LTFU compared with White patients. These findings complement studies by Khurana et al^11^ and Obeid et al,^14^, which reported higher LTFU rates among Black and Hispanic patients compared with White patients. Racial and ethnic minority groups, such as Black and Hispanic individuals, frequently encounter multiple barriers to consistent health care access, including discrimination, cultural differences, language barriers, socioeconomic challenges, and distrust of the health care system.^22,23^ Social determinants of health, in particular, have been shown to impede access to eye and vision care, as well as reduce treatment adherence.^24^ For example, a US study of patients with diabetes found that a higher burden of adverse social determinants of health was associated with lower utilization of eye care services.^25^ Addressing these disparities will require targeted interventions, such as culturally tailored patient education, improved access to health care services, and initiatives aimed at building trust between health care practitioners and historically underserved populations.

To address the observed risk factors for being LTFU, a multifaceted approach is necessary, targeting both patient-level and health care system–level challenges. At the patient level, personalized reminder systems, such as automated text messages, phone calls, or application-based notifications, can serve as regular prompts for upcoming appointments.^26^ These reminders would be particularly useful for asymptomatic patients who may not feel the immediate necessity to return for follow-up care. The patient’s preferred language could also be documented to ensure reminders are communicated in their preferred language and in a culturally sensitive manner. For patients living far from care centers, transportation assistance programs could alleviate LTFU rates by offering subsidized transport or hospital shuttle services to make it easier for patients to attend scheduled visits.^27^ Similarly, aligning appointment schedules with public transportation availability or arranging group transport for patients from the same geographic area could enhance attendance rates. Additionally, it would be beneficial for transportation services to liaise with clinician offices to confirm appointments and other relevant details, facilitating a unified continuity of care. Culturally and linguistically tailored patient education is another critical component of reducing LTFU rates, particularly for racial and ethnic minority populations who may face additional barriers. Simple, jargon-free educational materials available in multiple languages could help improve patients’ understanding of PDR and DME.^28^ Artificial intelligence chatbots, with their ability to enhance readability and offer clinically relevant insights on DR, could play a key role in accurately translating these educational materials and making them interpretable for patients.^29,30^ Furthermore, employing community health workers from similar cultural backgrounds can help bridge trust gaps between health care practitioners and racial and ethnic minority communities. Another promising approach is to strengthen collaborations between ophthalmologists and primary care practitioners (PCPs). Given that PCPs often have more regular contact with patients, they are well positioned to reinforce the need for follow-up with ophthalmology. Additionally, having an integrated EMR system across specialties could allow for improved monitoring of patients at risk of being LTFU. Multidisciplinary care teams could also hold patients accountable for their appointments through follow-up phone calls from nurses or case managers.^31^ Implementing these targeted strategies could substantially improve adherence to follow-up care and ultimately improve visual outcomes for patients with PDR or DME.

### Limitations

This study has some limitations. It is possible that some patients may have sought continuing care from another retina specialist without notifying their treating retinal specialist; however, this information was not accessible. Moreover, the use of data from tertiary care academic points of care in Canada’s most populous city may limit the generalizability of our findings to rural centers. Nonetheless, our sample size of 2961 patients with DR over a 10-year period represents a relatively robust and diverse sample size compared with similar analyses in this setting.^7,9^ Limited EMR data precluded our ability to account for additional variables that may have influenced patient follow-up and treatment adherence, such as chronic comorbidities, hemoglobin A_1c_ levels, concomitant medications, socioeconomic status, accessibility of health care services, and the patient’s level of understanding regarding their disease. In the Canadian health care system, a referral to an ophthalmologist is required from a PCP, walk-in clinic physician, emergency department physician, optometrist, or other physician; however, our analysis did not account for the source of patient referrals. Data pertaining to race and ethnicity were estimated using the validated R package wru; however, these findings must be interpreted with caution, given risks of misclassification.^32^ Our analysis was also unable to capture external influences pertaining to patients’ reasons for being LTFU, which is crucial for understanding patient adherence.

## Conclusions

Overall, our retrospective cohort study found that the odds of patients with DR being LTFU at 2 academic tertiary centers were significantly greater among males, patients living farther from the point of care, patients who were Black or Hispanic, patients initially receiving PRP (vs IVIs), those with a good baseline BCVA, as well as those with fewer follow-up appointments and a lower IVI burden. It is imperative for clinicians to appreciate the complex interplay of factors associated with higher LTFU rates, which will inform strategies targeted at reducing the burden and frequency of patient nonadherence in this setting.

## Notes

Supplement 1. **eTable 1.** Analysis of Interaction Terms in Loss to Follow-Up Multivariable Regression Model **eTable 2.** Sensitivity Analysis of One-Year Exposure Period Starting at Initial Treatment

Supplement 2.
Data Sharing Statement
