# Supplementary material for: Loss to Follow-Up in Patients With Proliferative Diabetic Retinopathy or Diabetic Macular Edema
Source: JAMA Netw Open. 2024 Dec 13;7(12):e2450942. doi: 10.1001/jamanetworkopen.2024.50942 (PMC11645645; doi:10.1001/jamanetworkopen.2024.50942)
Supplement: Supplement 1. — eTable 1. Analysis of Interaction Terms in Loss to Follow-Up Multivariable Regression Model eTable 2. Sensitivity Analysis of One-Year Exposure Period Starting at Initial Treatment [file jamanetwopen-e2450942-s001.pdf]

Supplemental Online Content

Huang RS, Naidu SC, Mihalache A, et al. Loss to follow-up in patients with proliferative diabetic retinopathy and diabetic macular edema. *JAMA Netw Open*. 2024;7(12):e2450942. doi:10.1001/jamanetworkopen.2024.50942

eTable 1. Analysis of Interaction Terms in Loss to Follow-Up Multivariable Regression Model
eTable 2. Sensitivity Analysis of One-Year Exposure Period Starting at Initial Treatment

This supplemental material has been provided by the authors to give readers additional information about their work.

© 2024 Huang RS et al. *JAMA Network Open.*

eTable 1. Analysis of Interaction Terms in Loss to Follow Up Multivariable Regression Model

	Adjusted OR	95% CI	P Value
Baseline VA X Treatment Type			
≤20/40, IVI	*Reference*		
20/50-20/200, IVI	1.84	(0.60-5.63)	0.286
20/50-20/200, PRP	1.07	(0.18-6.32)	0.944
>20/200, IVI	1.86	(0.54-7.12)	0.258
>20/200, PRP	2.65	(0.51-13.7)	0.244
Age X Number of Visits in 1st year			
<65 years, <6 visits	*Reference*		
65-74 years, ≥6 visits	1.11	(0.62-2.00)	0.721
75-84 years, ≥6 visits	1.09	(0.58-2.23)	0.787
≥85 years, ≥6 visits	1.03	(0.45-2.36)	0.947
Distance X Number of Visits in 1st year			
<20 km, <6 visits	*Reference*		
20-34 km, ≥6 visits	0.84	(0.44-1.60)	0.593
35-49 km, ≥6 visits	0.75	(0.38-1.49)	0.411
50-200 km, ≥6 visits	0.96	(0.43-2.18)	0.927
>200 km, ≥6 visits	0.72	(0.33-1.57)	0.405

VA: visual acuity, OR: odds ratio, CI: confidence interval.
Interaction terms were added one at a time to the multivariable regression model for LTFU consisting of the following variables: age, sex, race, distance, baseline BCVA, baseline DME, treatment at the first visit, treatment type at the first visit, number of visits in the first year, number of IVIs in the first year, and number of PRP sessions in the first year.

© 2024 Huang RS et al. *JAMA Network Open.*

eTable 2. Sensitivity Analysis of One-Year Exposure Period Starting at Initial Treatment

	No LTFU vs LTFU					
	Unadjusted OR	95% CI	P Value	Adjusted OR*	95% CI	P Value
Initial treatment type						
IVI	*Reference*			*Reference*		
PRP	2.74	(1.95-4.39)	<0.001	2.45	(1.70-4.06)	<0.001
Number of visits in exposure period						
<6	*Reference*			*Reference*		
≥6	0.68	(0.53-0.90)	0.010	0.69	(0.56-0.95)	0.018
Number of IVI in exposure period						
<6	*Reference*			*Reference*		
≥6	0.35	(0.15-0.70)	<0.001	0.38	(0.18-0.75)	<0.001
Number of PRP in exposure period						
<4	*Reference*			*Reference*		
≥4	1.59	(1.11-2.07)	0.011	1.50	(1.08-1.97)	0.023

IVI: intravitreal injection, LTFU: lost to follow up, OR: odds ratio, CI: confidence interval.
Multivariable regression model was adjusted for all predictors with $P<.2$ on univariable analysis, including: age, sex, race, distance, baseline BCVA, baseline DME, treatment at the first visit, treatment type at the first visit, number of visits in the first year, number of IVIs in the first year, and number of PRP sessions in the first year. Patients with missing, unknown, or indeterminate values for any covariate were excluded from the multivariable regression model (n=2,779 included in the multivariable model).

© 2024 Huang RS et al. *JAMA Network Open.*
