# Supplementary material for: Loss to Follow-Up in Patients With Proliferative Diabetic Retinopathy or Diabetic Macular Edema
Source: JAMA Netw Open. 2024 Dec 13;7(12):e2450942. doi: 10.1001/jamanetworkopen.2024.50942 (PMC11645645; doi:10.1001/jamanetworkopen.2024.50942)
Supplement: Supplement 2. — Data Sharing Statement [file jamanetwopen-e2450942-s002.pdf]

Data Sharing Statement

Huang. Loss to Follow-Up in Patients With Proliferative Diabetic Retinopathy or Diabetic Macular Edema. *JAMA Netw Open*. Published December 13, 2024.
doi:10.1001/jamanetworkopen.2024.50942

Data
Data available: No
